# Acetyl and butyryl cholinesterase inhibitory sesquiterpene lactones from *Amberboa ramosa*

**DOI:** 10.1186/1752-153X-7-116

**Published:** 2013-07-10

**Authors:** Muhammad Ibrahim, Tahir Farooq, Nusrat Hussain, Amjad Hussain, Tahsin Gulzar, Iqbal Hussain, Muhammad Sajid Akash, Fouzia Sultana Rehmani

**Affiliations:** 1 Department of Applied Chemistry, Government College University Faisalabad, Faisalabad, Pakistan; 2 International Centre for Chemical and Biological Sciences, HEJ Research Institute of Chemistry, University of Karachi, Karachi 75270, Pakistan; 3 Department of Botany, Government College University Faisalabad, Faisalabad, Pakistan; 4 Institute of Pharmacology, Toxicology and Biochemical Pharmaceutics, College of Pharmaceutical Sciences, Zhejiang University, Hangzhou, P.R. China; 5 Department of Chemistry, University of Karachi, Karachi 75270, Pakistan

**Keywords:** Amberbin C, *Amberboa ramosa*, Compositeae, Acetyl cholinesterase, Butyryl cholinesterase

## Abstract

**Background:**

Alzheimer’s disease (AD) is characterized by a progressive memory loss that leads to a profound emotional disturbance in later stages. As no safe and effective drug is yet available for the treatment of AD, secondary metabolites from plants may be instrumental in meeting this challenge. Keeping in view this point we evaluated sesquiterpenes of medicinal plant *Amberboa ramosa* for their cholinesterase inhibitory activity.

**Results:**

Four sesquiterpene lactones have been isolated from the ethyl acetate soluble fraction of *Amberboa ramosa*. In which one compound Amberbin C (**1**) was found to be new while other three Amberin (**2**), Amberbin A (**3**), and Amberbin B (**4**) were previously reported ones. The structures of the isolated compounds were elucidated using different spectroscopic techniques. Isolated compounds were tested for their inhibitory potential against acetyl cholinesterase and butyryl cholinesterase enzymes. All compounds showed excellent inhibitory activities against acetyl cholinesterase and butyryl cholinesterase.

**Conclusions:**

A new sesquiterpene lactone has been isolated and fully characterized, the sesquiterpene lactones from *Amberboa ramosa* showed good inhibitory activities against acetyl cholinesterase and butyryl cholinesterase enzymes, this study indicated that sesquiterpene lactone can become interesting lead molecules in drug development against Alzheimer’s disease (AD).

## Background

Cholinesterases (ChE) form an important class of enzymes intimately connected with the nervous system. Acetylcholine (ACh) was first synthesized in 1867. In 1906 it was detected in the adrenal gland of human tissue as a neurotransmitter, which transmits signals from one nerve cell to another. Cholinesterases ChE inactivate acetylcholine by hydrolyzing it into choline and acetic acid. Cholinesterase inhibitor increases the availability of ACh for nerve cell communications [1]. Alzheimer’s disease (AD) is characterized by a progressive memory loss that leads to a profound emotional disturbance in later stages. The disease is accompanied by dysfunctions in cholinergic neurotransmission of the central nervous system [2]. Hence, cholinesterase inhibitors may act as potential leads in the discovery of therapeutics for such nervous system disorders.

As the anti cholinesterase activity of chloroform soluble fraction from *Amberboa ramosa* has previously been reported [3], keeping in view this point we evaluated the guaianolides (sesquiterpenes) isolated from chloroform soluble fraction of *Amberboa ramosa* for their cholinesterase inhibitory activity. The genus *Amberboa* belongs to the family Compositeae and comprises of six species. *Amberboa ramosa* is an annual herb which belongs to genus *Amberboa* and family Compositeae. It is mainly found in Pakistan and India. *Amberboa ramosa* has tonic, aperient, febrifuge, deobstruent, cytotoxic and antibacterial activities [4]. Literature survey revealed that triterpenoids, flavonoids, steroids and sesquiterpene lactones have previously been reported from this species [4,5]. In this study we have isolated four guaianolides (sesquiterpenes) out of which Amberbin C (**1**) was identified as a new compound while other three Amberin (**2**) [6], Amberbin A (**3**) and Amberbin B (**4**) [7] were previously been reported from the same source. All structures were elucidated by using different spectroscopic techniques like UV, IR, EI-MS, 1D and 2D NMR techniques.

## Results and discussion

**Figure 1:**
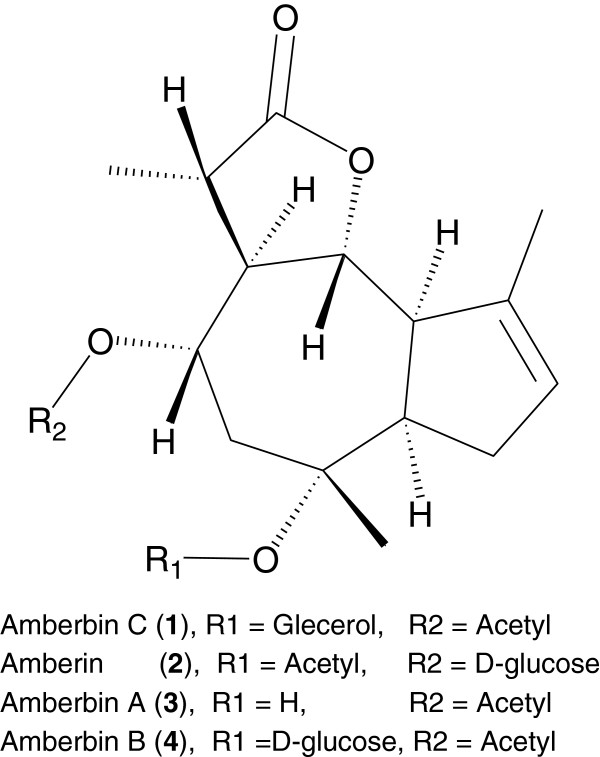
Structures of compounds 1–4.

Amberbin C (Figure 1) was isolated as colorless crystals with [*α*]20 D +51 (*c* = 0.02, CHCl_3_) and mp 141–142°C. The HR-EI-MS exhibited a [*M*+] peak at *m/z* 382.1997 corresponding to the molecular formula C_20_H_30_O_7_ (calcd. for C_20_H_30_O_7_; 382.1992), which indicated six degrees of unsaturation. Further prominent peaks at m/z 364.2, 308.2, 339.2 and 324.2 represented the losses of [MH_2_O]+, [M-C_3_H_6_O_2_] + and [M-COCH_3_-CH_3_]+, respectively (Figure 1).

**Figure 2:**
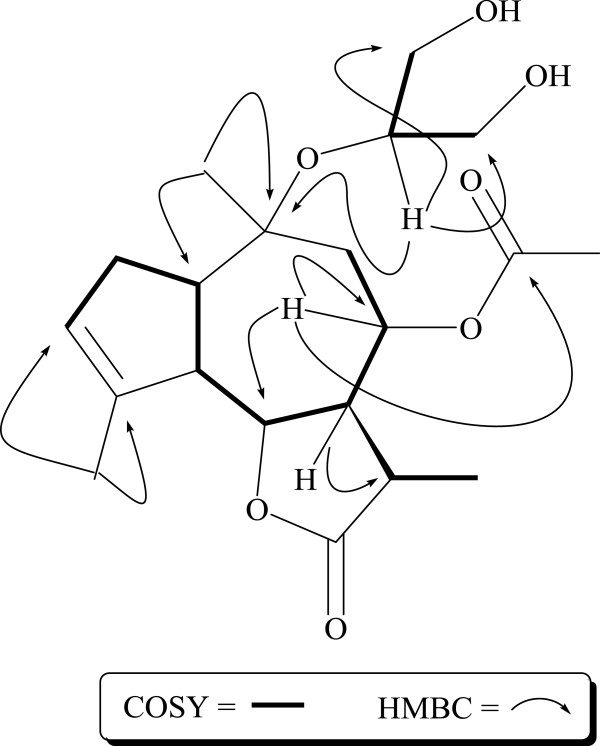
Important COSY and HMBC correlations.

The IR spectrum showed characteristic absorption bands for γ-lactone and ester groups at 3410 (OH), 1750 (lactone), 1730 (ester) and 1655 (C = C), respectively. In the UV spectrum moderate absorption bands between 196–202 nm further indicated the presence of γ-lactone moiety [8]. Further spectral data showed close agreement with a guaianolide type sesquiterpene [9-11]. The 1H-NMR spectrums showed signals for a trisubstituted double bond at *δ* 5.40. It also showed signals for the oxymethine protons at *δ* 5.07 (ddd, *J* = 8.3, 6.0, 5.5 Hz) and 4.25 (dd, *J* = 10.0, 8.3 Hz) (see Table 1). The latter was assigned to the proton geminal to the lactone oxygen atom [12]. It showed ^1^H-^1^H correlations to the vicinal protons at *δ* 2.90 (dd, *J* = 9.0, 8.8 Hz) and *δ* 2.85 (ddd, *J* = 10.0, 9.5, 8.3 Hz) which could subsequently be assigned to H-5 and H-7, respectively. The larger coupling constants suggested *trans*-diaxial disposition among H-5, H-6 (*β*) and H-7, providing conclusive evidence for an *α* orientation of both H-5 and H-7. The coupling pattern of the proton signals for H-1 and H-9 supported the guaianolide structure [13]. The entire sequence of protons attached to the guaianolide skeleton was established by correlation spectroscopy (COSY) and spin decoupling experiments (Figure 2). Irradiation of the H-5 proton at *δ* 2.90 simplified the double doublet of H-6 at *δ* 4.25 into a double and the doublet of double doublets of H-1 into a doublet doublet. Irradiation of H-7 proton at *δ* 2.85 simplified the doublet of quartet at *δ* 2.50 into a quartet. Irradiation of H-11 proton at *δ* 2.50 simplified the doublet of the methyl group at *δ* 1.24 into a singlet confirming the presence of a methyl group at C-11. Irradiation of H-3 at *δ* 5.40 turned the double doublets at *δ* 2.24 (H-2*α*) and 2.35 (H-2*β*) into doublets. The signal at 1.79 could be assigned to methyl protons at C-15 and further confirmed by ^*2*^ *J* and ^*3*^ *J* correlations with C-4 (*δ* 144.0), C-3 (*δ* 126.8) and C-5 (*δ* 55.0). The O-acetyl group was assigned to C-8, on the basis of strong HMBC correlation between oxymethine proton of C-8 at *δ* 5.07 and the carbonyl carbon at *δ* 181.0 as shown in Figure 2. The glycerol moiety could be assigned to C-10 based on its lower frequency shift compared to unsubstituted guaianolides [7], and supported by HMBC correlations between oxymethine proton of C-18 at *δ* 3.64 and C-10 at *δ* 81.1 (Figure 2). The signals at *δ* 3.59 (2H), 3.48 (2H) were assigned to four diastereomeric protons of glycerol moiety. These protons showed COSY and HMBC correlations with H-18 (*δ* 3.64) and C-18 (*δ* 73.9) (Figure 2). It further confirmed the presence of glycerol moiety. The ^13^C-NMR spectrum (Broad band and DEPT) showed signals for 20 carbons comprising of four methyls, four methylene, eight methine and four quaternary carbons. The low frequency region showed four signals at *δ* 181.0, 172.1, 144.0 and 126.8, which could be assigned to O-acetyl, lactone ester and trisubstituted olefinic carbons. One oxygenated quaternary and two oxygenated methine carbons resonated at *δ* 81.1, 75.4 and 82.3 respectively. The position of substituents was confirmed by HMQC, HMBC and COSY experiments.

**Table 1:** ^**1**^**H- and **^**13**^**C-NMR for compound 68; CD**_**3**_**OD soln.; δ in ppm, *J *in Hz**

**Position**	***δ *(H)**^**a**^**)**	***δ *(C)**^**b**^**)**	**Multiplicity**
1	2.80 ddd (9.0, 8.5, 8.0)	53.0	CH
2	2.35 dd (15.5, 5.0)	34.0	CH_2_
	2.24 dd (9.0, 8.0)		
3	5.40 brd s	126.8	CH
4	-	144.0	C
5	2.90 dd (9.0, 8.8)	55.0	CH
6	4.25 dd (10.0, 8.3)	82.3	CH
7	2.85 ddd (10.0, 9.5, 8.3)	52.3	CH
8	5.07 ddd (8.3, 6.0, 5.5)	75.4	CH
9	1.86 dd (12.3, 5.5)	42.8	CH_2_
	2.38 dd (12.3, 6.0)		
10	-	81.1	C
11	2.50 dq (11.0, 5.8)	42.1	CH
12	-	172.1	C
13	1.24 d (6.9)	15.7	CH_3_
14	1.27 s	26.1	CH_3_
15	1.79 s	17.7	CH_3_
16	-	181.0	C
17	2.05 s	21.3	CH_3_
18	3.63-3.65 m	73.86	CH
19	3.59 d	64.4	CH_2_
	3.56 d		
20	3.52 d	64.4	CH_2_
	3.48 d		

^a^) Recorded at 400 MHz; ^b^) Recorded at 100 MHz.

**Figure 3:**
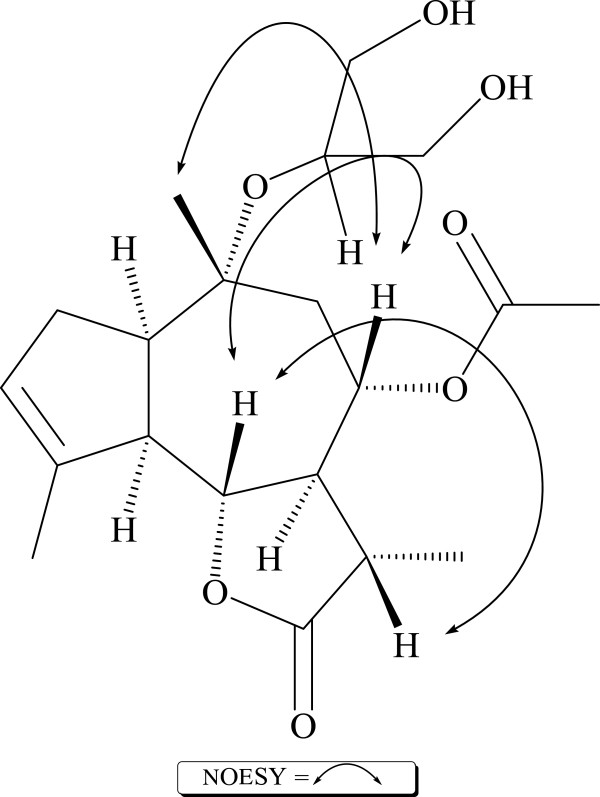
Important NOESY correlations.

The relative stereochemistry at various chiral centers of amberbin C (**1**) were assigned through NOESY experiments (Figure 3), which revealed *trans*/anti/*cis*-fusion of the α-methyl-γ-lactone moity, the seven member ring at C-7 and C-6, the five member ring at C-5 and C-1 [14-16]. The cis-fusion of guaianoloid is also proven by the strong correlation between the H-1 at *δ* 2.80 and H-5 at *δ* 2.90. The α-orientation of the acetate group at C-8 could also be deduced through strong interaction in NOESY experiment between the oxymethine proton attached to C-8 resonating at *δ* 5.07 and H-6 at *δ* 4.25. Thus, the structure of amberin C (**1**) could be assigned as 8*α*-Acetoxy-10*α*-(1, 3-dihydroxy-2-propyl)-1 *α* H, 5*α* H, 6*β* H, 7*α* H, 11 *β* H, 11*α*-methylguaia-3-enolide.

### Biological evaluation

The Compound (**1**) and (**2**) showed good inhibitory potential towards acetyl cholinesterase. Compound (**2**) also showed potent activity against butyryl cholinesterase. Compound (**3**), and (**4**) showed significant activity against acetyl cholinesterase and potent activity against butyryl cholinesterase (see Table 2). Compound (**1**) also showed moderate butyryl cholinesterase inhibitory activity. All four compounds share similar basic skeleton, they differ only due to the substituents present at C-8 and C-10. Compound **3** which have O-acetyl group at C-8 and OH group at C-10 showed moderate inhibitory potential towards both cholinestrease enzymes. Compound **4** only differs from Compound **3** due to the presence of an α-D-glucose group at C-10, the introduction of glucose moeity significantly increases the cholinestrease inhibitory activity. While in case of compound **2** which have glucose moeity at C-8 and O-acetyl at C-10 a significant decrease in acetylcholinestrease inhibitory activity occures, while butrylcholinestrease inhibitory activity was almost the same. Simillarly in case of compound **1** which have glycerol moeity at C-10 significant anti-acetylcholinestrease activity was observed. On the basis of these results we can conclude that the effect of substituents at C-10 is more important towards acetylcholinestrease enzyme, mostly activity enhances with the presence of substituents.

**Table 2:** ***In vitro *anticholinesterase activities of compounds 1–4**

		***IC***_***50 ***_**(μM) ± SEM**^**a**^	
**S. No**	**Compounds**	**AChE**	**BChE**
1	Amberbin C (**1**)	1.1 ± 0.08	17.9 ± 0.05
2	Amberin (**2**)	17.5 ± 0.01	2.7 ± 0.02
3	Amberbin A (**3**)	8.6 ± 0.15	4.8 ± 0.15
4	Amberbin B (**4**)	0.91 ± 0.015	2.5 ± 0.15
5	Galanthamine^b^	0.5 ± 0.01	8.2 ± 0.02
6	Eserine^b^	0.04 ± 0.0001	0.82 ± 0.001

^a^ Standard error of the mean of five assays; ^b^ positive control used in the assays.

## Conclusions

A new sesquiterpene lactone has been isolated and fully characterized, the sesquiterpene lactones from *Amberboa ramosa* showed good inhibitory activities against acetyl cholinesterase and butyryl cholinesterase enzymes, this study indicated that sesquiterpene lactone can become interesting lead molecules in drug development against Alzheimer’s disease (AD).

### Experimental

#### General experimental procedures

Melting points were determined on a Gallenkemp apparatus and are uncorrected. IR spectra were measured on a JASCO 302-A spectrophotometer with KBr cells. UV spectra were obtained on a Hitachi UV-3200 spectrophotometer. Optical rotations were measured on a JASCO DIP-360 polarimeter and the 1D and 2D NMR spectra were recorded on a Bruker AMX-400 Spectrometer operating at 400 MHz for ^1^H and 100 MHz for ^13^C. Electron impact (EI) mass spectra were recorded on JEOL JMS-HX-110 and Varian MAT-311-A mass spectrometers. The HR-ESI-MS was recorded on a Jeol JMS 600H instrument. Silica gel (230–400 mesh, E. Merck, Darmstadt, Germany) was used for column chromatography. All reagents were obtained from Sigma Aldrich Chemical sand used without further purification. The redistilled and de-ion-ized water was used in all experiments. The spectrophotometer, Spectra max 384 from molecular devices USA is used for inhibition protocol.

#### Plant material

The whole plant of *Amberboa ramosa* Jafri (Compositae) was collected in June 2002, from Malir district of Karachi (Pakistan) and identified by Dr. Surraiya Khatoon, Plant Taxonomist, Department of Botany, University of Karachi, where a voucher specimen (no. KU 312 b) has been deposited.

#### Extraction and isolation

The shade dried plant material (8 kg) was extracted three times with methanol at room temperature. Solvents were evaporated through vacuum distillation. The condensed and crude methanolic extract (217 g) was partitioned between n-hexane and water. The water soluble fraction was further fractionated into chloroform, ethyl acetate and n-butanol soluble fraction. The column chromatography of the EtOAc soluble sub-fraction (90 g) over silica gel, using a mixture of n-hexane/EtOAc (collecting 200 ml for each), afforded six major fractions A (n-hexane: EtOAc 8:2), B (n-hexane: EtOAc 6.5:3.5), C (*n*-hexane: EtOAc 5.5:4.5) D (n-hexane: EtOAc 4:6), E (n-hexane: EtOAc 3:7) and Fraction F (100% EtOAc).

**Sub-fraction A:** It was subjected to vacuum liquid chromatography (VLC) using the solvent gradient from *n*-hexane- EtOAc to afford two sub-fractions AA and AB. The sub-fraction AA obtained from *n-*hexane-EtOAc (8:2) was further purified by column chromatography over silica gel eluting with *n*-hexane-EtOAc (8.5:1.5) to furnish compound **3** (13.5 mg).

**Sub-fraction E:** It was subjected to column chromatography over silica gel using mixtures of *n*-hexane-EtOAc (3:7) as eluent to obtain two major sub-fractions EA - EB. The sub-fraction EA was subjected to further column chromatography over silica gel eluting with *n*-hexane- EtOAc (3.5:6.5) as eluent to give compound **1** (11 mg), and *n*-hexane- EtOAc (4:6) to give compound **2** (7.8 mg). The sub-fraction EB was subjected to successive column chromatography over silica gel eluting with *n*-hexane- EtOAc (3.2:6.8) solvent system to yield compound **4** (9.4 mg).

#### Enzyme inhibitory assays

*In vitro* cholinesterase inhibition assay and determination of *IC*_*50*_ Acetylcholinesterase (electric eel EC 3.1.1.7), butyrylcholinesterase (horse serum E.C. 3.1.1.8), acetylthiocholine iodide, butyrylthiocholine chloride, 5, 5′-dithiobis [2-nitrobenzoic-acid] (DTNB) and galanthamine were purchased from Sigma (St. Louis, MO, USA). Buffer and other chemicals were of analytical grade. Acetylcholinesterase and butyrylcholinesterase inhibiting activities were measured by a slightly modified spectrophotometric method [17]. Acetylthiocholine iodide and butyrylthiocholine chloride were used as substrates to assay acetylcholinesterase and butyrylcholinesterase, respectively. 5, 5′-Dithiobis [2-nitrobenzoic-acid] (DTNB) was used for the measurement of cholinesterase activity. 140 μL of (100 mM) sodium phosphate buffer (pH = 8.0), 10 μL of DTNB, 20 μL of test compound solution and 20 μL of acetylcholinesterase or butyrylcholinesterase solution were mixed and incubated for 15 min (25°C). The reaction was then initiated by the addition of 10 μL acetylthiocholine or butyrylthiocholine, respectively. The hydrolysis of acetylthiocholine and butyrylthiocholine was monitored by the formation of the yellow 5-thio-2-nitrobenzoate anion as the result of the reaction of DTNB with thiocholine, released by the enzymatic hydrolysis of acetylthiocholine and butyrylthiocholine, respectively, at a wavelength of 412 nm (15 min). Test compounds and control were dissolved in EtOH. All the reactions were performed in triplicate in 96-well micro plates and monitored in a Spectra Max 340 (Molecular Devices, USA) spectrometer. Estimation of *IC*_*50*_ values: The concentrations of test compounds which inhibited the hydrolysis of substrates (acetylthiocholine and butyrylthiocholine) by 50% (*IC*_*50*_) were determined by monitoring the effect of increasing concentrations of these compounds in the assays on the inhibition values. The *IC50* values were then calculated using the EZ-Fit Enzyme Kinetics program (Perrella Scientific Inc., Amherst, USA).

## Competing interests

All the authors declare that they have no competing interests.

## Authors’ contributions

FSR supervised all the research work. MI and AH carried out isolation and purification of sesquiterpenes, NH interpreted nmr spectra and made a significant contribution to acquisition of data, analysis, drafting of the manuscript. IH carried out isolation, purification and characterization of the constituents. MI and TG facilitate in research work. MSHA carried out *In vitro* anticholinesterase activities. All authors read and approved the final manuscript.
